# Eight weeks of resistance training in conjunction with glutathione and L-Citrulline supplementation increases lean mass and has no adverse effects on blood clinical safety markers in resistance-trained males

**DOI:** 10.1186/s12970-018-0235-x

**Published:** 2018-06-27

**Authors:** Paul Hwang, Flor E. Morales Marroquín, Josh Gann, Tom Andre, Sarah McKinley-Barnard, Caelin Kim, Masahiko Morita, Darryn S. Willoughby

**Affiliations:** 1 Exercise and Biochemical Nutritional Lab, Department of Health, Human Performance, and Recreation, Baylor University, Waco, TX USA; 2 Department of Kinesiology, University of Louisiana Monroe, Monroe, LA USA; 3 Department of Health, Exercise Science, and Recreation Management, University of Mississippi, University, MS USA; 4 Department of Health and Kinesiology, University of South Alabama, Mobile, AL USA; 5 Function Research Group, Healthcare Products Development Center, Kyowa Hakko Bio Co., Ltd., 2, Miyukigaoka, Tsukuba, Ibaraki, Japan

**Keywords:** L-citrulline, Glutathione, Resistance training, Muscle strength, Body composition

## Abstract

**Background:**

Supplementation of combined glutathione (GSH) with L-citrulline in response to a single bout of resistance exercise has been shown to increase plasma nitric oxide metabolites, nitrite and nitrate and cyclic guanosine monophosphate (cGMP), which may play a role in muscle protein synthesis. As a result, in response to resistance training (RT) these responses may establish a role for GSH + L-citrulline to increase muscle mass.

This study attempted to determine the effects of an 8-week RT program in conjunction with GSH (Setria®) + L-citrulline, L-citrulline-malate, or placebo supplementation on lean mass and its association with muscle strength. The secondary purpose was to assess the safety of such supplementation protocol by assessing clinical chemistry markers.

**Methods:**

In a randomized, double-blind, placebo-controlled design, 75 resistance-trained males were randomly assigned to ingest GSH + L-citrulline (GSH + CIT), L-citrulline-malate, or cellulose placebo daily while also participating in 8 weeks of RT. The full dose of each supplement was delivered in capsules that were identical in weight, size, shape, and color. Participants completed testing sessions for body composition and muscle strength before and after 4 and 8 weeks of RT and supplementation. Venous blood samples were obtained before and after 8 weeks.

**Results:**

Leg press was increased with RT but was not significantly different between groups (*p* > 0.05); however, bench press strength was not increased with RT (*p* > 0.05). There were no significant changes in total body mass, fat mass, or total body water during 8 weeks of RT and supplementation. Lean mass increased in both GSH + CIT when compared to PLC; however, the increase was significant only after 4 weeks. Lean mass and strength were positively correlated (*p* < 0.05) in GSH + CIT, but not CIT-malate or PLC. Neither RT nor supplementation had any significant effects on blood clinical chemistry variables (*p* > 0.05).

**Conclusion:**

Compared to PLC, supplementation of GSH + CIT during resistance training increased lean mass after 4 weeks of RT and was positively associated with muscle strength. However, after 8 weeks of RT there were no significant differences in any of the measured variables.

## Background

L-citrulline is a nonessential amino acid and is co-produced with nitric oxide (NO) as an end-product of nitric oxide synthase (NOS). Unlike L-arginine, it can bypass hepatic metabolism and is transported to the kidneys where it is directly converted into L-arginine [1]. As a result, exogenous L-citrulline supplementation represents an alternative to increase the amount of L-arginine provided to NOS. Compared to L-arginine, an advantage to supplementation with L-citrulline is that intestinal catabolism of L-citrulline is limited since it is not metabolized by arginases, and the activity of arginosuccinate synthase is low in enterocytes [2]. Furthermore, L-citrulline is not extracted from the systemic circulation for hepatic clearance [3].

*S*-nitrosothiols possess the capacity to act as NO donors and also facilitate NO transfer reactions [4]. Low molecular weight thiols such glutathione (GSH) have been shown to up-regulate the NO-pathway and form *S*-nitrosoglutathione (GSNO) upon interaction with NO [5]. Glutathione has been shown to stabilize low levels of NO and to slowly release NO due to GSNO conversion. Furthermore, GSH has been shown to react with newly-created free radicals, thereby protecting NO from oxidative damage and potentiating the effectiveness of NO [6].

Glutathione possesses the ability of GSH to stabilize and slowly release NO, thereby protecting it for oxidative damage. Therefore, we tested this concept in vivo where we showed 7 days of L-citrulline combined with GSH supplementation to increase plasma L-citrulline and L-arginine levels. In addition, in response to a single bout of resistance exercise, plasma NO metabolites, nitrate and nitrite, and cyclic guanosine monophosphate (cGMP) were elevated 30 min following exercise [7]. These results are noteworthy as it has been shown that NOS activity is necessary for calcium-induced activation of the Akt pathway. Furthermore, NO appears to influence protein kinase B (Akt) signaling though a cGMP/phosphoinositide 3-kinase- (PI3K) dependent pathway [8], which is the primary pathway for up-regulating translation initiation required for muscle protein synthesis (MPS).

L-citrulline has been shown to increase NO and vasoactivity [7] and potentially up-regulate MPS [8]. In addition, L-citrulline has been shown to have a positive impact of exercise performance [9]. As a result, supplementation of L-citrulline has generated interest in the exercise/sports nutrition arena based on its ability to produce ergogenic responses, particularly during acute, single-bout exercise scenarios. For instance, L-citrulline malate supplementation was effective at increasing the number of repetitions for selected resistance exercise movements in untrained [9] and resistance-trained males [10, 11]. However, what may be much more impactful is the potential ergogenic effect of L-citrulline after a longer period (days or weeks) of supplementation. However, there appear to be no studies involving longer-term (e.g., 8–12 weeks) periods of supplementation. Presently, there are studies which have evaluated the effectiveness of L-citrulline or citrulline malate after periods of 7 and 16 days. Shorter-term (7 days) L-citrulline malate supplementation has been shown to augment skeletal muscle power output due to a greater oxidative energy turnover and a lower pH-to-power ratio [12]. It has also been shown to result in a lower adenosine triphosphate (ATP) cost of muscle force production [13], which suggests that shorter-term supplementation of L-citrulline malate might improve skeletal muscle metabolism.

Seven days of L-citrulline supplementation at a daily dose of 2.4 g was shown to significantly increase plasma nitrite and nitrate, citrulline, and arginine. Moreover, in response to a 4-km cycling time trial, L-citrulline was shown to significantly reduce time trial performance [14]. In response to 16 days of supplementation with 3.4 g/d, L-citrulline significantly increased plasma citrulline, arginine, and nitrite levels, and also increased muscle oxygenation; however, there were no superior effects on time-to-exhaustion during high-intensity exercise [15].

Taken together, we hypothesized that the role of L-citrulline as an ergogenic aid in response to longer-term RT may be synergistically enhanced by the combination with GSH. As a result, the effects of RT and GSH + L-citrulline supplementation on body composition and muscle performance, and its potential as an ergogenic aid in humans, is in need of elucidation. Therefore, the primary purpose of this study was to determine the effects of an 8-week RT program in conjunction with daily, orally-delivered GSH + L-citrulline, L-citrulline-malate, or placebo supplementation on body composition and muscle performance. The secondary purpose was to assess the safety of such supplementation protocol by assessing whole blood and serum clinical chemistry markers.

## Methods

### Experimental approach

Using a random number generator (www.random.org), in a double-blind, placebo-controlled design, 75 resistance-trained males between the ages of 18 and 35 were randomly assigned to ingest L-citrulline malate, L-citrulline + GSH, or placebo while also participating in 8 weeks of resistance training. Participants completed 3 testing sessions where body composition and muscle performance were assessed and venous blood was obtained prior to RT and supplementation and after 8 weeks of RT and supplementation.

### Participants

Seventy-five apparently healthy, resistance-trained [regular, consistent resistance training (i.e. thrice weekly) for at least one year prior to the onset of the study], males between the ages of 18–35 and a body mass index between 18.5–25 kg/m^2^ completed the double-blind study. Enrollment was open to men of all ethnicities. Only participants who were non-smokers, were considered as low risk for cardiovascular disease with no contraindications to exercise as outlined by the American College of Sports Medicine (ACSM), who had not consumed any nutritional supplements (excluding multi-vitamins) 3 months prior to the study, and were free from orthopedic problems that would inhibit participation in upper- and lower-body resistance training exercises were allowed to participate. All participants provided written informed consent and were cleared for participation by passing a mandatory medical screening. All eligible subjects signed university-approved informed consent documents and approval was granted by the Institutional Review Board for the Protection of Human Subjects of Baylor University and Research Ethical Review Committee of Kyowa Hakko Kirin Co., Ltd. Additionally, all experimental procedures involved in the study conformed to the ethical consideration of the Declaration of Helsinki.

### Body composition testing procedures

At each of the 3 testing sessions, total body mass (kg) was determined on a standard dual beam balance scale (Detecto Bridgeview, IL). Fat mass and lean mass were determined using DEXA (Hologic Discovery Series W, Waltham, MA, Apex System Software Version 4.6.0.2). Quality control calibration procedures was performed on a spine phantom (Hologic X-CALIBER Model DPA/QDR-1 anthropometric spine phantom) and a density step calibration phantom prior to each testing session. Previous studies in our lab have shown the accuracy of DEXA for body composition to be ±3.8% as assessed by direct comparison with hydrodensitometry and scale weight. Total body water was determined with bioelectrical impedance spectroscopy (TBF-410GS, Tanita Inc., Arlington Heights, IL, USA) using a low energy, high frequency current (500 micro amps at a frequency of 50 kHz). Coefficients of variation from ongoing research from our lab over the course of several years has shown the accuracy of DEXA for body composition to be ±3.8, and 4.6% for total body water assessed using bioelectrical impedance spectroscopy.

### Muscle strength assessments

For the assessment of muscle strength, participants performed 1 repetition maximum (1-RM) tests on the free-weight bench press and angled leg press exercises [16] prior to the first dose of supplement and beginning of the resistance-training program and after 4 and 8 weeks RT and supplementation. Participants warmed-up by completing 10 repetitions at 50% of their total body mass. The participant rested for 1 min, and then completed 3 to 5 repetitions at 75% of their body mass. The weight was then increased conservatively, and the participant attempted to lift the weight for one repetition. If the lift was successful, the participant rested for 2 min before attempting the next weight increment. This procedure was continued until the participant failed to complete the lift. The 1-RM was recorded as the maximum weight that the participant could lift for 1 repetition. For the bench press, a requirement for a successful attempt was for the bar to lightly touch the chest during bar descent. For the angled leg press, range of motion (ROM) was set at 90°. This angle was established goniometrically for each participant and a mark made on the leg press machine to insure this ROM was reached on each attempt for each participant. Based on our previous work, a goal of no more than five attempts was set for all 1-RM testing sessions [16]. All participants obtained their 1-RM within five, and the average (±SEM) attempts for all subjects over the two 1-RM testing sessions was 3.91 (±0.76).

### Dietary analysis

Participants were required to record their dietary intake for 4 consecutive days prior to each of the 3 testing sessions that occurred at prior to baseline and during weeks 4 and 8. The participants’ diets were not standardized and they were asked not to change their dietary habits during the course of the study. The 4-day dietary recalls were evaluated with the Food Processor dietary assessment software program (ESHA Research, Salem, OR) to determine the average daily amount of kilocalories and macronutrient consumption of fat, carbohydrate, and protein in the diet at three different time points during the study.

### Supplementation compliance

Supplementation compliance was monitored by participants returning empty containers of their supplement when they reported for their final 8-week testing session, and by completing weekly supplement compliance questionnaires.

### Supplementation protocol

L-Citrulline and GSH (Setria®) were obtained from Kyowa Hakko Bio Co., Ltd. (Tokyo, Japan). Participants were matched by total body mass and then randomly assigned a supplementation protocol, in double-blind fashion, consisting of the oral ingestion of either 200 mg/day of GSH + 2 g/day of L-citrulline [GSH + CIT (*n* = 25)], 2 g/day of L-citrulline-malate [CIT-malate (*n* = 25)], or 2.52 g/day of cellulose placebo (*n* = 25). Participants took their supplement one hour prior to exercise. On non-exercise days, participants took their supplement in the morning with breakfast. The full dose of each supplement was delivered in seven capsules that were identical in weight, size, shape, and color, and appropriately blinded (provided by Kyowa Hakko Bio Co., Ltd.)

### Resistance training protocol and volume determination

Participants engaged in a supervised, periodized 4-day per week resistance-training program split into two upper- and two lower-extremity workouts per week for a total of 8 weeks [16]. Prior to the workout, participants performed a standardized series of stretching exercises. The participants then performed an upper-body resistance-training program consisting of the bench press, lat pull, shoulder press, seated row, shoulder shrug, chest fly, biceps curl, triceps press down, and abdominal curl exercises twice per week. The lower-body program consisted of leg press, back extension, step up, leg curl, leg extension, heel raise, and abdominal crunch exercises, also performed twice per week. Participants performed 3 sets of 10 repetitions with as much weight as they could lift per set [typically 70–80% of the one repetition maximum (1-RM)]. Rest periods between exercises lasted 2 min. Resistance training was supervised and the training volume monitored by study personnel. RT volume was determined for the 8 weeks for the upper- and lower-body exercises, and was calculated as the number of sets x the number of repetitions x weight/set x the number of training sessions.

### Reported side effects from supplements

After week 4 and 8, participants reported by questionnaire whether they tolerated the supplement, supplementation protocol, and reported any medical problems/symptoms they encountered throughout the duration of the study.

### Venous blood sampling

Venous blood samples were obtained from the antecubital vein into a 10 ml collection tubes using a standard vacutainer apparatus into one tube for serum separation and one for whole blood. The serum separation tubes were allowed to stand at room temperature for 15 min, centrifuged for 10 min, and serum was removed and placed into a microfuge tube. Whole blood tubes and serum microfuge tubes were refrigerated and then picked up by a courier and outsourced for analysis (Quest Diagnostics, Waco, TX) within 2 h. A total of two blood samples were obtained at baseline and after week 8.

### Whole blood and serum clinical safety markers

Whole blood and serum samples were outsourced to an independent commercial laboratory for analysis (Quest Diagnostics, Waco, TX). Serum samples were assayed for general clinical chemistry markers [i.e., glucose, total protein, blood urea nitrogen (BUN), creatinine, BUN/creatinine ratio, uric acid, aspartate aminotransferase (AST), alanine aminotransferase (ALT), creatine kinase (CK), lactate dehydrogenase (LDH), gamma glutamyl transferase (GGT), albumin, globulin, sodium, chloride, calcium, carbon dioxide, total bilirubin, alkaline phosphatase, triglycerides, cholesterol, high density lipoprotein (HDL), and lower density lipoprotein (LDL)]. Whole blood samples were assayed for standard cell blood counts with percentage differentials [i.e., hemoglobin, hematocrit, platelet, red blood cell counts, mean corpuscular volume (MCV), mean corpuscular hemoglobin (MCH), mean corpuscular hemoglobin concentration (MCHC), red blood cell distribution width (RDW), white blood cell counts (neutrophils, lymphocytes, monocytes, eosinophils, basophils).

### Statistical analysis

Data are presented as means ± standard error of the mean (SEM). Data was analyzed by a repeated-measures analysis of variance (ANOVA). Statistical comparisons between the baseline and corresponding post-test values were analyzed using Bonferroni’s test for multiple comparisons following ANOVA. A correlation among lean mass and muscle strength was analyzed with Pearson’s correlation coefficient test. A *p*-value of less than 0.05 was considered to indicate significance. Statistical analysis was performed with Statcel software for Windows (Version 2, OMS Publishing, Inc. Saitama, Japan) and the Ystat Statistical Program File (Igaku Tosho Shuppan, Tokyo, Japan).

## Results

### Consort information

A total of 84 individuals were recruited and screened; however, 3 were ineligible due to 1 not being resistance-trained and 2 had previously taken nutritional supplements that were included in the exclusionary criteria. There were 81 participants that began the study, but 6 withdrew prior to completion. Of these, 2 dropped out due to injuries sustained not related to resistance training and 4 due to schedule conflicts that prohibited them from remaining compliant to the study protocol; 75 participants (25 in each group) completed the study. Of the 75, the mean ± SEM age, height, and % body fat was 20.47 ± 2.42 yr., 70.02 ± 2.54 in., and 15.55 ± 5.13%, respectively. In addition, 48% were Caucasian, 36% were Asian, 8% were Hispanic, and 8% were African-American. For supplementation compliance, participants in placebo, CIT-malate, and GSH+ CIT all reported a compliance rate of 100%.

### Body composition

In regard to body composition (Table 1), there were no significantly different changes in total body mass, fat mass, or total body water over the course of the 8-week resistance training and supplementation period. Total body mass and fat mass for GSH + CIT was significantly less than placebo at week 8 (*p* < 0.05); however, this was due to the fact that this group was also significantly less than placebo at baseline and was not due to the experimental intervention.

**Table 1:** Body mass, lean mass, fat mass, total body water throughout the study period

Variable	Baseline	Supplementation (week)	
		4	8
Body Mass (kg)			
Placebo	81.60 ± 3.0	82.10 ± 3.04	82.30 ± 2.96
CIT-malate	79.20 ± 2.50	79.50 ± 2.52	80.10 ± 2.42
GSH + CIT	73.60 ± 1.74*	74.60 ± 1.69	74.60 ± 1.73*
Lean Mass (kg)			
Placebo	59.92 ± 10.42	59.93 ± 10.33	59.81 ± 9.81
CIT-malate	59.43 ± 8.47	59.59 ± 8.46	59.84 ± 7.93
GSH + CIT	55.70 ± 7.28	56.46 ± 6.91*	56.20 ± 6.93
Fat Mass (kg)			
Placebo	13.10 ± 1.17	13.70 ± 1.19	14.00 ± 1.20
CIT-malate	11.20 ± 1.04	11.50 ± 0.92	11.90 ± 0.96
GSH + CIT	9.90 ± 0.71	10.30 ± 0.71	10.60 ± 0.69*
Total Body Water (kg)			
Placebo	48.20 ± 1.31	48.20 ± 1.32	48.40 ± 1.28
CIT-malate	47.90 ± 1.14	48.10 ± 1.16	48.40 ± 1.14
GSH + CIT	45.00 ± 0.89	45.50 ± 0.89	45.60 ± 0.89
Δ Lean Mass (kg)			
Placebo		0.01 ± −0.09	−0.11 ± 0.61
CIT-malate		0.16 ± − 0.01	0.41 ± − 0.54
GSH + CIT		0.76 ± − 0.37	0.5 ± − 0.35
Δ Total Body Water (kg)			
Placebo		0.0 ± 0.01	0.2 ± 0.03
CIT-malate		0.2 ± 0.02	0.3 ± 0.0
GSH + CIT		0.5 ± 0.0	0.6 ± 0.0
Net Δ LM-TBW (kg)			
Placebo		0.01 ± − 0.10	−0.79 ± 0.58
CIT-malate		0.04 ± 0.08	0.11 ± − 0.54
GSH + CIT		0.26 ± 0.37	0.10 ± − 0.35

Mean ± SEM, *Significantly different from Placebo (*p* < 0.05), Δ = change from baseline, Net Δ = difference in the change in lean mass and total body water from baseline, *LM* lean mass, *TBW* total body water

### Lean mass

**Fig. 1:**
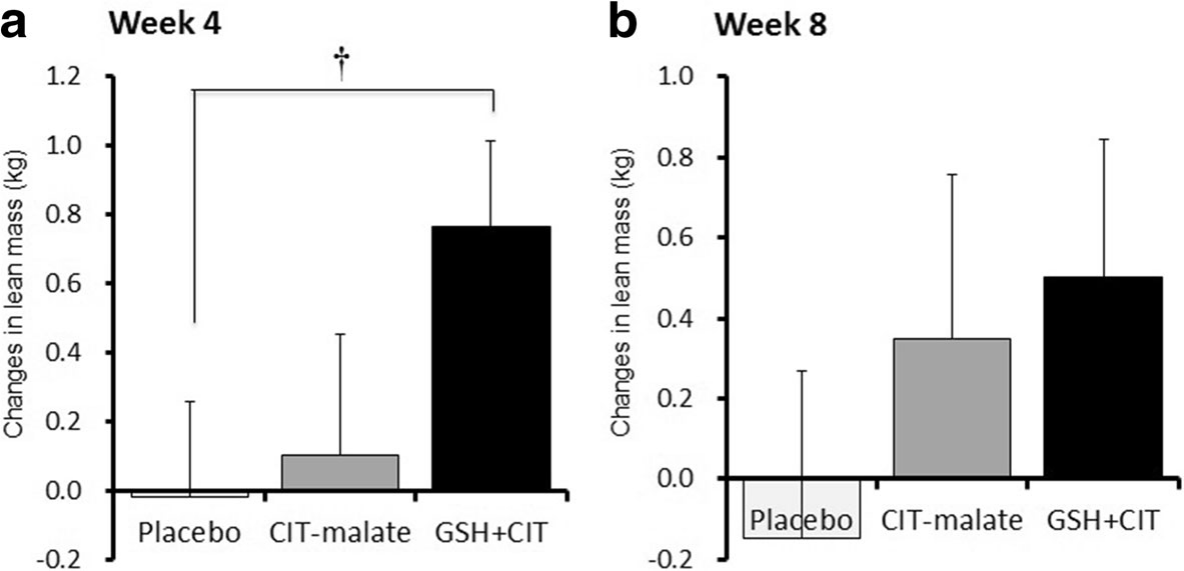
Delta changes relative to baseline (PRE) in lean mass in each group at 4 weeks (**a**) and 8 weeks (**b**) following heavy resistance training and supplementation. Mean ± SEM, † Significantly different from Placebo (*p* < 0.05)

As can be seen in Table 1 and Fig. 1, changes in GSH + CIT were significantly greater than PLA after 4 weeks (*p* < 0.05) but failed to maintain statistical significance after 8 weeks (*p* > 0.05). After 8 weeks, lean mass for GSH + CIT was still greater than PLC; however, this difference was not significantly different (*p* > 0.05).

### Muscle strength

For muscle strength, there were no significant differences for bench press strength (*p* > 0.05) over the course of the 8 weeks. For leg press strength, all three groups significantly increased strength over the course of the 8 weeks (*p* < 0.05); however, there were no significant differences between groups (*p* > 0.05) (Table 2).

**Table 2:** Muscle strength for the bench press and leg press exercises throughout the study period

Variable	Baseline	RT and Supplementation (week)	
		4	8
Bench Press (kg)			
Placebo	91.27 ± 22.63	93.56 ± 21.59	97.27 ± 25.42
CIT-malate	93.72 ± 21.93	96.81 ± 21.97	100.63 ± 26.30
GSH + CIT	88.54 ± 19.24	92.81 ± 18.59	95.45 ± 23.53
Leg Press (kg)			
Placebo	397.81 ± 69.84	440.43 ± 78.71*	470.77 ± 101.12*†
CIT-malate	389.72 ± 104.56	429.36 ± 115.85*	469.45 ± 126.49*†
GSH + CIT	342.54 ± 74.99	394.54 ± 71.72*	423.36 ± 97.65*†

Mean ± SEM. Bench press and leg press strength expressed in kg. *Significantly different from baseline (*p* < 0.05); †Significantly different from week 4 (*p* < 0.05)

### Relationship between lean mass and muscle strength

**Fig. 2:**
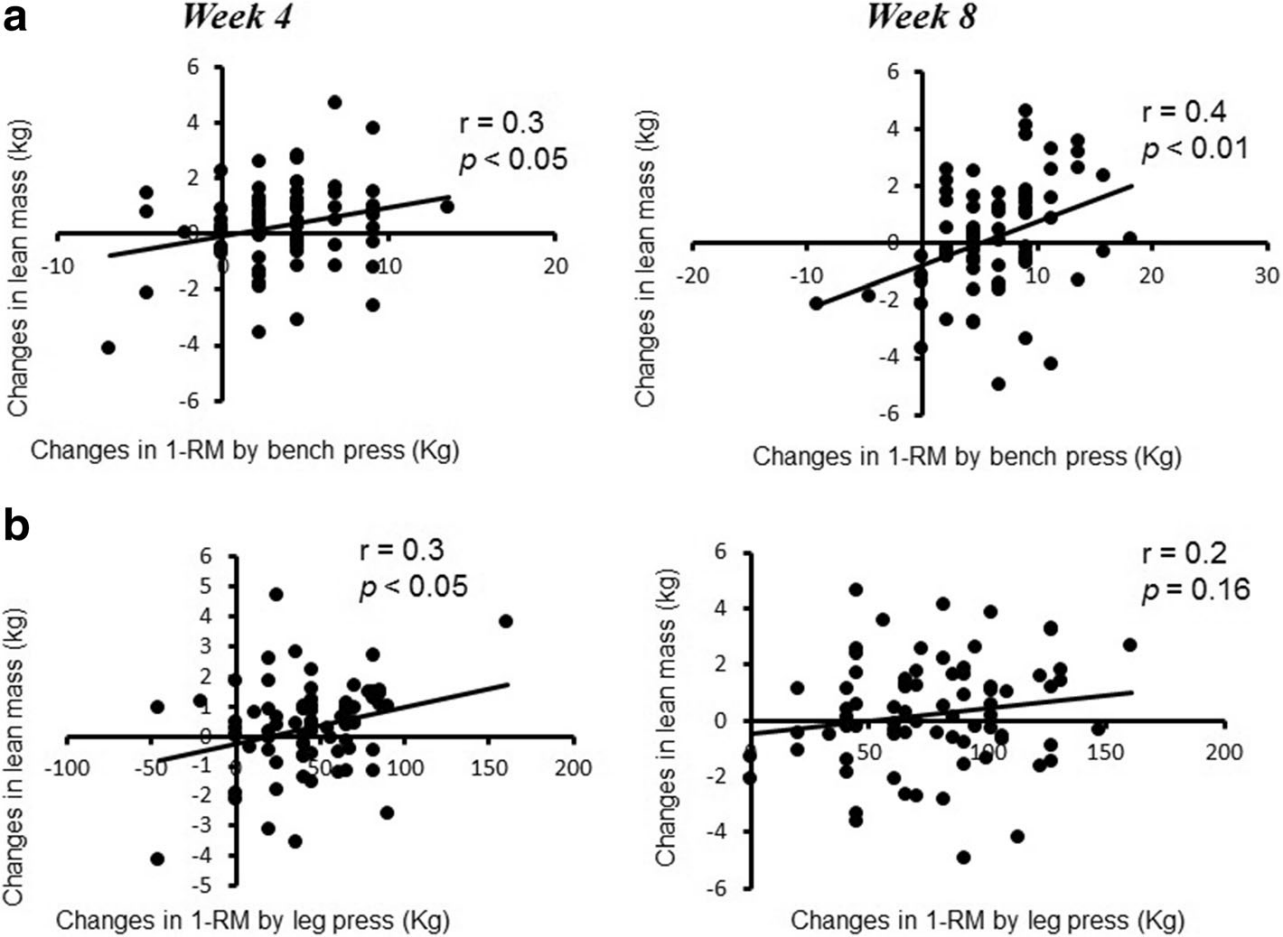
Relationship of changes in lean mass with 1RM bench press (**a**) and 1RM leg press (**b**) for all three groups at weeks 4 and 8. For the bench press, significant correlations were observed at 4 and 8 weeks (*p* < 0.05). However, for the leg press a significant correlation was only observed at week 4 (*p* < 0.05). Mean ± SEM

**Fig. 3:**
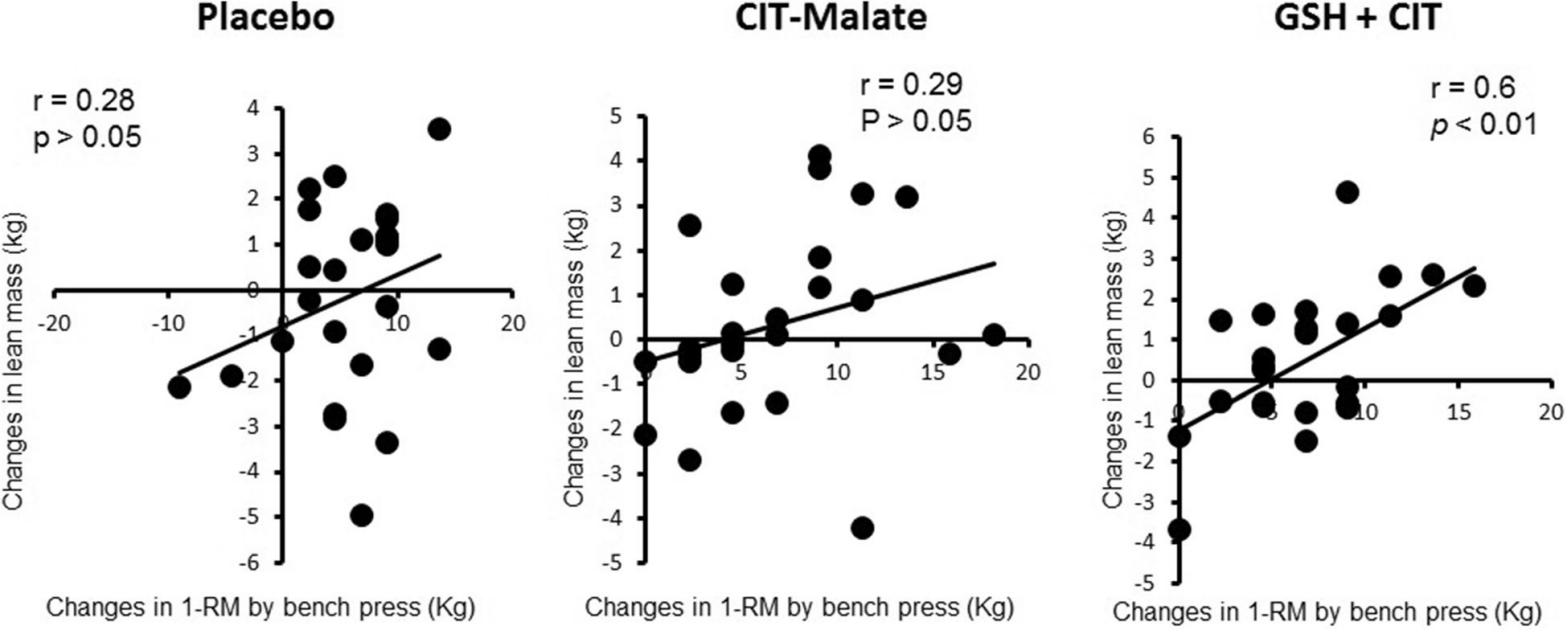
Relationship of changes in lean mass with 1RM bench press in each group throughout the study period. A significant correlation was observed only for GSH + CIT (*p* < 0.05). Mean ± SEM

Figure 2 shows the relationship between muscle mass and strength at week 4 and 8 for the bench press and leg press exercises, respectively. In regard to lean mass and bench press strength, there was a significant relationship observed at week 4 (*r* = 0.3, p < 0.05) and week 8 (*r* = 0.4, *p* < 0.01). However, for lean mass and leg press strength a significant relationship was observed only at week 4 (r = 0.3, p < 0.05). Furthermore, a significant correlation between lean mass and strength was only observed in GSH + CIT (Fig. 3) which suggests that GSH + CIT supplementation, but not CIT-malate, increased lean mass independent of significant increases in strength.

### Dietary composition and resistance training volume

For dietary composition, there were no significant main effects or interaction in the content of macronutrients or in total daily calories (*p* > 0.05). By the three different assessment points, this indicates that none of the dietary variables were significantly changed over the course of the 8 weeks for either group, and that neither group differed from the other (Table 3).

**Table 3:** Dietary composition throughout the study period

Variable	Baseline	RT and Supplementation (week)	
		4	8
Total kcal/kg			
Placebo	28.26 ± 10.18	26.98 ± 8.13	25.61 ± 6.88
CIT-malate	27.85 ± 8.54	25.61 ± 7.02	24.61 ± 6.94
GSH + CIT	30.90 ± 7.66	30.14 ± 9.59	29.26 ± 8.88
Protein (g/kg)			
Placebo	1.49 ± 0.57	1.44 ± 0.58	1.43 ± 0.45
CIT-malate	1.43 ± 0.49	1.41 ± 0.58	1.25 ± 0.36
GSH + CIT	1.74 ± 0.61	1.57 ± 0.62	1.57 ± 0.75
Carbohydrate (g/kg)			
Placebo	3.06 ± 1.30	2.81 ± 0.95	2.81 ± 0.91
CIT-malate	3.05 ± 1.18	2.65 ± 1.01	2.70 ± 1.05
GSH + CIT	3.08 ± 0.64	3.29 ± 0.95	3.10 ± 0.82
Fat (g/kg)			
Placebo	1.29 ± 0.48	1.39 ± 0.60	1.14 ± 0.37
CIT-malate	1.23 ± 0.46	1.28 ± 0.51	1.13 ± 0.36
GSH + CIT	1.52 ± 0.54	1.51 ± 0.67	1.49 ± 0.73

Mean ± SEM. Each value represents the average amount for the 4-day recall and expressed relative to total body mass. No significant differences between groups for any of the dietary variables (*p* > 0.05)

Resistance training volume was expressed relative to total body mass. For upper-body, the resistance training volumes were 4541.26 ± 978.71, 4279.77 ± 1176.06, and 4796.63 ± 1468.31, respectively, for placebo, CIT-malate, and GSH + CIT. For lower-body, the volumes were 6159.98 ± 1212.64, 6229.04 ± 1577.92, and 6426 ± 2279.50 for placebo, CIT-malate, and GSH + CIT, respectively. There was no significant difference in training volume between groups (*p* > 0.05) over the course of the 8 weeks for exercises involving the upper- and lower-body.

### Serum and whole blood clinical safety markers

As can be seen in Table 4, there was a significant main effect for Group that existed for urea nitrogen and absolute basophils (*p* < 0.05) where GSH + CIT was less than PLC. However, this was due to the fact that the baseline values for GSH + CIT were both less than PLC and was not due to the experimental intervention. There were no significant main effects for Time and the interaction for Group x Time noted (*p* > 0.05) suggesting none of the clinical chemistry variables were significantly changed over the course of the 8 weeks for either group, and that neither group differed from the other.

**Table 4:** Changes in blood biochemical and hematological markers before and after resistance training and supplementation

Variable	Placebo		CIT-malate		GSH + CIT	
	Baseline	8 weeks	Baseline	8 weeks	Baseline	8 weeks
Glucose (mg/dl)	93.3 ± 2.7	93.1 ± 3.4	97.5 ± 3.4	101.8 ± 3.9	93.9 ± 1.7	93.5 ± 2.8
Total protein (g/dl)	7.3 ± 0.1	7.2 ± 0.1	7.4 ± 0.1	7.2 ± 0.1	7.4 ± 0.1	7.3 ± 0.1
Urea nitrogen (mg/dl)	18.6 ± 0.9	16.9 ± 0.6	16.8 ± 0.8	16.9 ± 0.7	16.1 ± 0.7*	15.4 ± 0.5
Creatinine (mg/dl)	1.1 ± 0.03	1.0 ± 0.02	1.1 ± 0.03	1.1 ± 0.04	1.0 ± 0.02	1.0 ± 0.02
Bilirubin (mg/dl)	0.6 ± 0.05	1.0 ± 0.3	0.7 ± 0.08	0.6 ± 0.05	0.7 ± 0.1	0.6 ± 0.07
AST (IU/l)	25.9 ± 1.9	23.4 ± 1.4	23.8 ± 1.5	21.3 ± 1.3	25.6 ± 3.2	21.8 ± 1.8
ALT (IU/l)	26.0 ± 2.4	25.8 ± 2.8	24.9 ± 2.4	22.9 ± 1.8	28.3 ± 7.9	20.0 ± 1.4
Albumin (g/dl)	4.9 ± 0.04	4.7 ± 0.05	4.9 ± 0.05	4.7 ± 0.05	4.8 ± 0.05	4.7 ± 0.04
Albumin/Globulin	2.0 ± 0.04	1.9 ± 0.05	2.0 ± 0.07	1.9 ± 0.06	1.9 ± 0.05	1.8 ± 0.05
ALP (IU/l)	71.0 ± 3.4	71.3 ± 4.4	74.9 ± 4.5	73.0 ± 4.5	71.5 ± 4.4	71.0 ± 3.5
WBC (× 10^3^/μl)	5.5 ± 0.3	5.3 ± 0.3	6.2 ± 0.3	5.7 ± 0.3	6.1 ± 0.3	5.7 ± 0.3
RBC (× 10^6^/μl)	5.1 ± 0.08	5.1 ± 0.07	5.1 ± 0.07	5.1 ± 0.06	5.1 ± 0.05	5.1 ± 0.06
Hb (g/dl)	15.3 ± 0.2	15.3 ± 0.2	15.1 ± 0.2	15.0 ± 0.1	15.1 ± 0.2	15.0 ± 0.2
Platelet (× 10^3^/μl)	222.3 ± 7.3	203.2 ± 7.1	218.2 ± 10.9	212.4 ± 8.0	237.3 ± 14.2	223.5 ± 12.2
Red cell distribution width	13.1 ± 0.1	13.2 ± 0.2	13.3 ± 0.1	13.5 ± 0.4	13.2 ± 0.1	13.1 ± 0.1
Absolute Neutrophils (μl^− 1^)	3042.8 ± 217.4	2877.7 ± 300.0	3652.2 ± 306.1	3292.4 ± 318.9	3548.3 ± 235.3	3312.7 ± 259.4
Absolute Lymphocytes (μl^− 1^)	1864.1 ± 87.4	1784.3 ± 93.9	2032.1 ± 131.4	1902.7 ± 85.5	1956.6 ± 110.3	1780.1 ± 84.8
Absolute Monocytes (μl^− 1^)	411.4 ± 39.6	457.7 ± 35.3	371.8 ± 25.6	370.5 ± 30.7	445.8 ± 39.1	402.3 ± 38.1
Absolute Eosinophil s (μl^− 1^)	185.5 ± 30.1	209.7 ± 36.2	130.5 ± 16.6	312.8 ± 79.5	155.6 ± 30.2	256.9 ± 43.8
Absolute Basophils (μl^− 1^)	33.8 ± 4.4	20.9 ± 1.9	31.2 ± 3.3	26.8 ± 3.2	22.8 ± 2.7*	22.1 ± 2.6

Mean ± SEM, *Significantly different from Placebo (*p* < 0.05). *AST* aspartate aminotransferase, *ALT* alanine aminotransferase, *ALP* alkaline phosphatase, *WBC* white blood cell, *RBC* red blood cell, *Hb* hemoglobin

## Discussion

This study sought to determine the effects of an 8-week RT program in conjunction with daily, orally-delivered GSH + CIT, CIT-malate, or placebo supplementation on body composition, the association between lean mass and muscle strength, and whole blood and serum clinical chemistry markers in resistance-trained men. We found that none of the three supplement interventions had any significant effect on fat mass, total body water, and blood clinical chemistry variables. However, we did observe GSH + CIT to undergo significant increases in lean mass compared to placebo after 4 weeks. Additionally, a significant correlation between lean mass and muscle strength was observed in GSH + CIT, but not PLC and CIT-malate.

We did observe GSH + CIT to undergo significant increases in lean mass compared to placebo after 4 weeks. Additionally, a significant correlation between lean mass and muscle strength was observed in GSH + CIT, but not PLC and CIT-malate.

Based on the results of previous studies showing increases in muscle performance [9–11, 17] in response to a single 8-g dose of L-citrulline malate, it can be assumed that longer durations of L-citrulline supplementation might bestow ergogenic effects. Case in point, 7 days of L-citrulline supplementation at a daily dose of 6 g significantly increase plasma citrulline, arginine, and nitrite levels, VO_2_ kinetics, in response to moderate intensity (70% VO_2_ peak) exercise performance [18]. Another study showed that 7 days of L-citrulline supplementation at a daily dose of 2.4 g significantly improved cycling time trial performance [14]. However, while 16 days of L-citrulline supplementation at a daily dose of 3.4 g significantly increased plasma citrulline, arginine, and nitrite levels, and increased muscle oxygenation during moderate-intensity (70% VO_2_ peak) exercise, there were no superior effects on time-to-exhaustion during high-intensity (90% VO_2_ peak) exercise. Although, the lack of impact on exercise performance in this study may have been due to the lower dose of L-citrulline compared to other studies [15].

In response to 8 weeks of L-citrulline supplementation and RT for body composition, our present results indicate that neither GSH + CIT or CIT-malate had any preferential and significant effect on total body mass, fat mass, and total body water; any changes that occurred were most likely due to the RT program. However, the data suggest that GSH + CIT increased lean mass over placebo after 4 weeks, and that a similar increasing tendency compared to PLC existed after 8 weeks. As indicated in Fig. 1, for GSH + CIT it should be noted that we observed a modest decrease in lean mass at 8 weeks compared to 4 weeks. Additionally, Fig. 3 shows some participants undergoing decreases in lean mass in all three groups during the study. Unfortunately, we are not able to provide a specific explanation for this response. However, it is possible this may have occurred since this group was eating less at week 8 than at baseline regarding kcal/kg and protein/kg. In lieu of within-participant differences that inherently exist in all studies with humans, this result may have been due to issues with effort exerted during the resistance training sessions, perhaps related to over-reaching and fatigue, differences in resistance training volume, supplementation compliance, and differences in dietary intake. However, our results show no significant differences between groups for any of these variables.

Excluding the inherent limitations that are known to exist with dietary self-reports, we expressed caloric intake relative to body mass, and based on the fact that there were no significant changes over the course of the 8-week study for the dietary variables, and that GSH + CIT did not consume more total calories or protein than the other two groups, dietary intake can likely be ruled out as a possible confounding variable for the increased lean mass. However, following baseline we only assessed dietary intake for 4 consecutive days prior to each of the two testing sessions at weeks 4 and 8 (8 out of 56 days) which constitutes a small portion of the dietary intake, approximately 86%, not accounted for during the study. Therefore, the role that dietary impact may have played on lean mass should be interpreted with caution.

We attempted to equate training volume and expressed it relative to body mass so that we could better determine any preferential effects provided by the supplements relative to the association between lean mass and muscle strength and showed there to be no differences between groups for RT volume. Interestingly, we did show that a significant relationship existed between lean mass and strength at week 4 and 8 for the bench press exercise. Regarding lean mass and leg press strength, however, there was a significant relationship observed only at week 4. More specifically, we observed that, only for GSH + CIT, the increase in muscle strength was significantly correlated to the increase in lean mass for this group.

Based on previous studies [8, 19, 20] it is conceivable that the increases in lean mass we observed for GSH + CIT in the present study could have occurred due to increases in muscle protein synthesis, and this could be linked to NO-induced increases in cGMP [19]. Even though we have yet to generate any specific data to support this statement, with our previous study [7] we did show that in response to a single bout of resistance exercise, the plasma NO metabolites, nitrate and nitrite, and cGMP were elevated 30 min following exercise when taking GSH + CIT. Regarding a possible sustained release of NO due to GSH + CIT, there are possible physiological benefits for having high NO levels at 30 min post-exercise relative to its impact on muscle protein metabolism and possible muscle performance in response to RT. For instance, it has been shown that NOS activity is necessary for calcium-induced activation of the Akt pathway (involved in translation initiation and subsequent muscle protein synthesis). Nitric oxide appears to influence Akt signaling though a cGMP/PI3K-dependent pathway [8], which is the primary pathway for up-regulating translation initiation and MPS. Similarly, NO seems to influence skeletal muscle function through effects on excitation-contraction coupling, myofibrillar function, perfusion, and metabolism. Another study showed that by using an agent to inhibit phosphodiesterase-5, that the augmentation of NO-cGMP signaling increased protein synthesis and reduced fatigue in human skeletal muscle [20]. In our previous study [7], GSH + CIT showed an improvement in cGMP activity suggesting that if this outcome was prevalent in the present study it could likely play a role in MPS and muscle performance when combined with longer-term RT. This suggests that a resistance exercise-related mechanism of inducing plasma NO, perhaps due to increased shear stress that triggered an up-regulation in NO-cGMP signaling along with a slow, sustained release of NO from GSH + CIT, may be a conceivable candidate for this response.

Considering the longer-term L-citrulline supplementation employed, we observed none of the whole blood and serum clinical chemistry markers to be negatively impacted by any of the three supplements. By the variables assessed, these results indicate that the oral ingestion of these supplements for a period of 8 weeks appears to be safe. In addition, none of the participants reported any adverse events associated with ingestion of the supplements.

Considering the typical sample size of 8–12 participants in each group for most studies with a similar experimental design, in the present study our sample size of 25 in each group can be considered a strength, rather than a limitation. Considering this, however, our study does possess several possible obvious limitations. The first limitation is a major issue and involves only using four-day dietary recalls for determining nutritional intakes prior to each of the three testing sessions, as it is possible that the information provided from the dietary intakes were not reflective of the nutritional intakes over the course of the study since we only assessed 8 out of the 56 days. Secondly, supplement compliance is a potential limitation. Even though participants returned the empty containers and self- reported their compliance to be 100%, it is possible that the information provided to study personnel was not accurate and reflective of the actual supplement compliance. Despite our confidence in the reliability and validity of our data, in lieu of these limitations, the results presented herein should be interpreted with some caution.

## Conclusion

In the present study, compared to PLC, supplementation of GSH + CIT during resistance training increased lean mass after 4 weeks of resistance training and was positively associated with muscle strength. However, after 8 weeks of resistance training there were no significant differences in any of the measured variables. However, more longer-term RT studies need to be conducted to generate a better understanding of its mechanisms of action. Based on the results presented herein, we conclude that, compared to placebo, supplementation of L-citrulline with GSH during resistance training increases lean mass in resistance-trained males.

## Abbreviations

1-RM: One-repetition maximum
ALT: Alanine aminotransferase
AST: Aspartate aminotransferase
ATP: Adenosine triphosphate
cGMP: Cyclic guanosine monophosphate
CK: Creatine kinase
GGT: Gamma glutamyl transferase
GSH: Glutathione
GSNO: *S*-nitrosoglutathione
LDH: Lactate dehydrogenase
MCH: Mean corpuscular hemoglobin
MCHC: Mean corpuscular hemoglobin concentration
MCV: Mean corpuscular volume
MPS: Muscle protein synthesis
NOS: Nitric oxide synthase
PI3K: Phosphoinositide 3-kinase
RDW: Red blood cell distribution width
RT: Resistance training
SEM: Standard error of the mean
